# Effect of double-induced on whey protein isolate nanoparticle formation and stabilized food-grade Pickering emulsions: Stability and gastrointestinal digestion

**DOI:** 10.1016/j.fochx.2025.102221

**Published:** 2025-02-06

**Authors:** Shenghua He, Yonghui Wang, Guanghui Li, Xueli Gao, Zhiyan Chen, Weiyun Guo, Jihong Huang

**Affiliations:** a Food and Pharmacy College, Xuchang University, Xuchang 461000, China; b Collaborative Innovation Center of Functional Food Green Manufacturing, Xuchang 461000, China

**Keywords:** Whey protein isolation, Nanoparticle, Food-grade, Pickering emulsion, Stability, Digestion properties

## Abstract

The effects of non-heat-induced and double-induced(heat-induced and Na^+^-induced) whey protein isolate nanoparticles (WPINs) at concentrations of C = 1 %, 2 %, and 5 % as well as oil fractions (φ = 0.1, 0.4, and 0.7) on the properties of food-grade Pickering emulsions (PEs) were systematically investigated. At a Na^+^ concentrations of 300 mM, the particle size of double-induced WPINs (290 μm) is significantly larger (*P* *<* 0.05) than that of non-heat-induced WPINs (210 μm). PEs stabilized by double-induced WPINs exhibited a significantly small particle size (*P* *<* 0.05) compared to those stabilized by non-heat-induced WPINs under identical oil fraction and WPIN concentration conditions. Additionally, PEs with φ = 0.7 stabilized by double-induced WPINs enhanced storage and thermal stability. However, both types of PEs exhibited freeze-thaw instability. PEs stabilized by double-induced WPINs showed a slower release rate of free fatty acid during gastrointestinal digestion. These findings highlight the promising application potential of WPINs in food-grade Pickering emulsion.

## Highlights

• Double-induced WPI nanoparticles (WPINs) exhibit significant potential to act as Pickering-type stabilizers.
• The Pickering emulsions stabilized by double-induced WPINs demonstrated excellent storage and thermal stability.
• Double-induced WPINs as Pickering-type stabilizers can effectively extend intestinal release of bioactive substances.

## 1 Introduction

The research and development of novel food-grade nanoparticle emulsifiers to replace traditional emulsifiers for stabilizing O/W Pickering emulsions (PEs) has aroused widespread interest across various fields, including food science, cosmetics, medicine, chemical industry, and materials science (Ning et al., 2020; Li et al., 2024; Li, Huang, et al., 2023; Li, Liu, et al., 2023; Naji-Tabasi et al., 2021). This interest is primarily attributed to the compatibility of PEs with food products and some remarkable features, such as extraordinary stability against coalescence, environmental tolerance, adjustable performance, low toxicity, and favorable delivery capability (Guo et al., 2024; Dickinson, 2010; Chevalier & Bolzinger, 2013; Berton & Schroën, 2015; Li et al., 2020). To date, there has been a renewed focus on the development and characterization of food-grade PE stabilizers from “biological sources,” owing to their potential applications in food formulations (Liu & Tang, 2016a,Liu & Tang, 2016b). Some researchers categorize food-grade PE particles into three main types: i) carbohydrate-type; ii) protein-type; and iii) miscellaneous. Notable examples include soy protein nanoparticles, whey protein nanoparticles, zein, and pea protein nanoparticles (Gupta & Rousseau, 2012; Liang & Tang, 2013; Xu et al., 2022). Protein-based particles are considered as the most promising food-grade PE stabilizers due to their unique features, which do not require chemical treatment and surface properties modification (Liu et al., 2017). A notable feature of these particles is their ability to absorb at the O/W interface, thereby forming a gel-like particle network between droplets that enhances emulsion stability (Liu & Tang, 2016a; Liu & Tang, 2016b; Liu & Tang, 2016c). Consequently, researchers are exploring both the cost-effectiveness and performances of food-grade PE stabilizers widely used in various sectors including food products, nutrition, dietary supplements, and medicine (Liu & Tang, 2016a; Liu & Tang, 2016b; Liu & Tang, 2016c; Rayner et al., 2012; Rayner et al., 2014; Ren et al., 2024; Ren et al., 2025). However, for these proteins to function effectively of food-grade PE stabilizers, they often require specific treatments. These include anti-solvent process (Joye & McClements, 2013), catalysis of transglutaminase (Li et al., 2024), pH-shift treatment (Sun et al., 2018), and protein-polysaccharide complex (Modiri-Dovom et al., 2024; Ren et al., 2023). Some studies have indicated that thermal treatment serves as a simple yet effective method for transforming plant and animal proteins into nanoparticles suitable for use as PE stabilizers (Liu & Tang, 2013). Heat treatment is known to cause protein structure changes and aggregations by formation of disulfide bonds, which can greatly promote the internal integrity of protein-based particles (Lam et al., 2014; Wang et al., 2012). For instance, thermal treatment of soy protein solutions can lead to the dissociation of protein subunits and the formation of soy protein nanoparticles. Results indicated that both heating temperature and protein concentration influence proteins aggregation (Keerati-U-Rai & Corredig, 2009; Cui et al., 2014). It has been reported that soy protein isolate (SPI) formed particle-aggregates upon heat-induced process and exhibited good surface activity for forming PEs with fine particles (Liu & Tang, 2014). A single heat treatment of protein makes it difficult to achieve the ideal effect. Besides the heating, the addition of divalent ions (Na^+^ and Ca^2+^) in protein solution is regarded as an effective method to induce aggregation of proteins and form protein-based particles since divalent ions can decrease the electrostatic repulse and result in particle aggregation in a high tendency by intermolecular disulfide bonds. However, to date, there have been relatively few reports on the combination of salt ion-induced process with heat-induced methods to form protein nanoparticles. Shi et al. (2017) reported that Ca^2+^ was utilized to induce and prepare the pea protein isolation (PPI) nanoparticles. Additionally, it has been reported that PPI microgel particles were prepared using transglutaminase as a cross-linker to stabilize high internal phase PE (Jiao et al., 2018). Although some studies have indicated that WPINs can be formed by various pathways and employed for emulsion stabilization, There remains a lack of systematic investigations into their stability such as storage stability, pasteurization stability and freeze-thaw stability and digestive properties of double-induced WPINs stbilized PEs. Thus, in the present work, whey protein isolation nanoparticles (WPINs) were formed through heat treatment and Na^+^ induced (double-induced), and the effect of Na^+^ concentrations on the properties of WPIN was systematically evaluated. Subsequently, PEs were then prepared through high-speed homogenizing flaxseed oil with WPINs water solution. The effects of WPINs concentrations and oil fractions on droplet size, zeta potential, microstructure, as well as stability of PEs were thoroughly investigated. Finally, the gastrointestinal digestive properties of PEs stabilized by different WPIN concentrations and special oil fractions (φ = 0.7) were examined. The article aims to provide a theoretical foundation and technical guidance for the application of food-grade PE stabilization using protein nanoparticles in food processing.

## 2 Materials and methods

### 2.1 Materials

Whey protein isolate (WPI, purity>90 %) was obtained from Shanxi Baichuan Biotechnology Co., Ltd. (Shanxi, China). Simulated gastric juice and simulated intestinal fluid were procured from Dongguan Chuangwei Testing Instrument Co., Ltd. Flaxseed oil was obtained from Zhejiang Jiusheng Camellia Technology Co., Ltd. (Zhejiang, China) and used to prepare PEs. All chemical reagents (purity>98 %) are required AR-grade. Ultrapure water was employed to prepare various solutions.

### 2.2 Preparation of WPINs

The method for the formation of WPINs was slightly modified based on the work of Liu and Tang (2013). A solution of WPI(8 wt%) was prepared, and NaCl was added to adjust the salt concentration to a range of 0–500 mM, respectively. The mixture was stirred at 200 rpm for 4 h and then allowed to stand overnight at 4 °C to ensure completely hydration of the protein. To inhibit microbial growth in the solution, 0.02 % sodium azide was incorporated. The pH of WPI solution was adjusted to 7.0 using a 1.0 M NaOH solution, followed by heating in a water bath at 85 °C for 30 min. Subsequently, an ice bath was employed to rapidly cool the solutions to room temperature (25 °C) within a span of 5 min.

### 2.3 Determination of particle size

The particle size of WPINs was measured by static light scattering using a Mastersizer 2000 (Mastersizer 2000, Malvern, UK). The particle size distribution and average particle size were measured after the sample was uniformly mixed and subsequently introduced to the sample cell (Wang et al., 2023). Various pH solutions were used to dilute samples to the desirable pH. The refractive index of the sample and water is 1.47 and 1.33, respectively. The average particle size of the samples is expressed by the volume- average particle size(D4.3), while the dispersion index (PDI) serves as a measure uniformity in the particle size distribution of the samples.

### 2.4 Determination of zeta potential

Zetasizer Nano ZS (Malvern Panalytical Ltd., NaNO-ZS ZEN3600, UK) was used to measure the surface potential of WPINs. Prior to measurement, the samples were diluted with aqueous solutions that mach the pH values and ion concentrations of the original samples. The samples were diluted 20 times. This approach aims to mitigate multiple scattering phenomena during the measurement process (Wang et al., 2023).

### 2.5 Preparation and properties of PEs

#### 2.5.1 Preparation of PEs

PEs with varying oil fractions (φ = 0.1, 0.4, and 0.7) were prepared by mixing suspensions of WPINs (Double-induced at 300 mM Na^+^ and 85 °C for 30 min) at various concentrations (C = 1 wt%, 2 wt%, and 5 wt%) in a glass vial. A homogenizer (T25 IKA-Ultraturrax digital, Germany) was employed to homogenize the mixtures at a speed of 10,000 rpm for a duration of 3 min, resulting in the formation of PEs. 0.02 % sodium azide was added to PEs to inhibit the microbial growth and then subjected to further experiments.

#### 2.5.2 Analysis of particle size and zeta-potential

A laser particle size analyzer (Mastersizer 2000, Malvern, UK) and a Zetasizer Nano ZS (Malvern Panalytical Ltd., NaNO-ZS ZEN3600, UK) were employed to assess the particle size and the zeta-potential of emulsion samples, respectively (Wang et al., 2023).

### 2.6 Rheological behavior of PEs

The rheological properties of the PEs were determined using a dynamic shear rheometer (DHR-1 rheometer, TA Instruments, Delaware, USA) equipped with a parallel plate (1.0 mm gap, 40 mm diameter) (Li et al., 2022). The moduli of the PEs were recorded in the frequency scan mode in the 10–100 rad/s range. The viscosity of the PEs was measured with shear rates increasing from 0.1 to 100 s^−1^. Measurements were performed at a constant strain amplitude of 1 % (in the linear viscoelastic region). All measurements were conducted at 25 °C.

### 2.7 Microstructure of PEs

Both an optical microscope (EX21, Ningbo) with a 40× objective magnification and a laser scanning confocal microscope (CLSM) (Zeiss, Germany), also equipped with a 40× objective magnification, were employed to observe the droplet morphology of PEs (Modiri-Dovom et al., 2024). For the optical microscope, 100 μL samples were dropped slightly on slides and covered glasses carefully with a coverslip to avoid bubble formation. In the case of CLSM, PEs with a special φ = 0.7 were stained with fluorescein isothiocyanate (FITC) and Nile red, The samples were then vortexed for 30 s and incubated in the dark for 10 min. Subsequently, 100 μL of the stained samples was gently applied onto slides and covered with coverslips. The excitation wavelengths for FITC and Nile red were set at 488 nm and 561 nm, respectively (Huang et al., 2021).

### 2.8 Stability of PEs

#### 2.8.1 Storage stability

The PEs were stored at 4 °C for 14 d, after which samples were taken every 2 days. The creaming index (CI) was employed to evaluate the creaming stability of the emulsions, defined as the height of the clear liquid in the emulsion expressed as a percentage of the total height of the emulsion (Ning et al., 2020).

#### 2.8.2 Thermal stability

The PEs were placed in a centrifuge tube and then heated in a water bath at 85 °Cfor 30 min. Subsequently, the samples were allowed to cool before analysis. The creaming index (CI) was employed to evaluate the creaming stability of the emulsions.

#### 2.8.3 Freeze-thaw stability

The PEs were transferred into a glass bottle and stored at −18 °Cfor 20 h. Subsequently, the samples underwent 3 thawing cycles at room temperature for 2 h. The stability of the PEs was determined immediately following each cycle (Li, Huang, et al., 2023; Li, Liu, et al., 2023). The creaming index (CI) was employed to evaluate the creaming stability of the emulsions.

The CI % were calculated by the following eq. (1):

(1) $$\mathrm{CI}\;\left(\%\right)=\frac{H_{e}}{H_{t}}\times 100$$

where H_e_ is the height of the clear liquid, and Ht is the total height of emulsions.

### 2.9 In vitro digestibility of PEs

The method for in vitro digestion of PEs was conducted following the protocol established by Wang et al. (2018). The simulated gastric juice (SGF) consists of 3.2 mg/mL pepsin, 0.7 % HCl, and 2 mg/mL NaCl. Initially, the pH of PEs was gradually lowered to approximately pH 2.0 by adding SGF solution at a flow rate of 1.5 mL/min using a pump, the whole gastric digestion was maintained a constant temperature of 37 °C at a water bath. The entire digestive process lasted for 2 h with samples taken every 30 min to measure potential charge.

Simulated intestinal fluid consists of 10 mM CaCl_2_, 150 mM NaCl, 5 mg/L Bile salt, and 1.6 mg/L trypsin (Wei et al., 2020). The simulated intestinal fluid was mixed with the previously digested gastric content (w:w 3: 1) and adjusted to pH 7.0 using a solution of 0.1 M NaOH. The pH value was controlled at this level through continuous addition of 0.1 M NaOH solution as needed to maintain stability throughout the experiment. Intestinal digestion occurred under constant temperature of 37 °C for 2 h, with sampling performed every 30 min to assess potential charge changes.

The content of free fatty acid release was calculated according to the following formula (2):

(2) $$\mathrm{FFA}\;\text{release}\;\left(\mu \;\mathrm{mol}/\mathrm{mL}\right)=\left(\frac{V_{\mathrm{NaOH}}\times C_{\mathrm{NaOH}}}{V_{\mathrm{IDM}}}\right)\;$$

where V_NaOH_ is the consumed volume (mL) of NaOH solution when the digestion time of simulated small intestinal is t; V_IDM_ is the volume of the intestinal digestive mixture (IDM) before intestinal digestion; C_NaOH_ is the molar concentration of NaOH solution.

### 2.10 Statistical analysis

The statistical analysis was performed by SPSS statistical software (version 23; SPSS Inc., Chicago, IL). All experiment was carried out three times. Statistical differences were assessed using Duncan's test and significant differences were accepted with *P* < 0.05.

## 3 Results and discussions

### 3.1 Effects of ionic strength and heat-induced on zeta potential, particle size and dispersion index (PDI) of WPINs

**Fig. 1:**
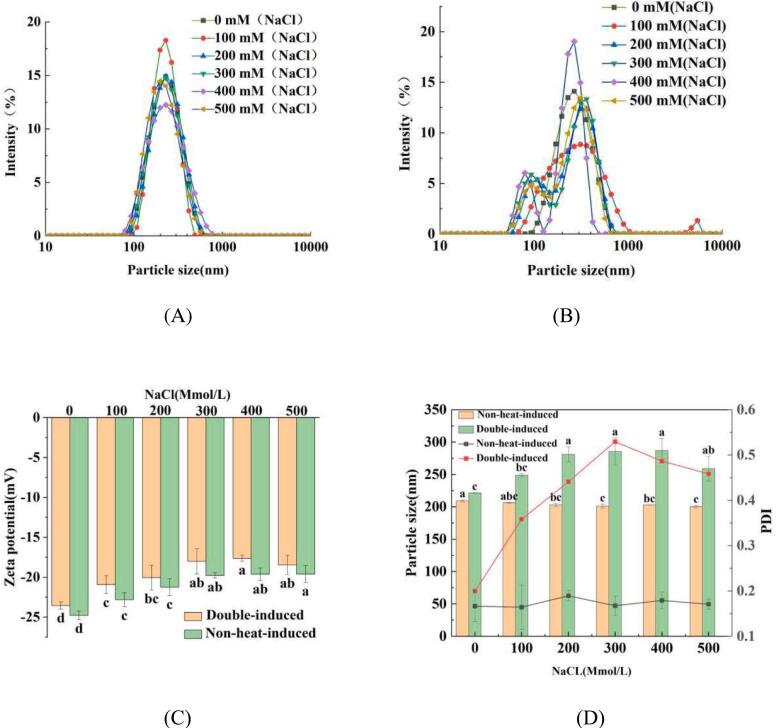
Effects of non-heat-induced (A) and double-induced (B) treatments on the particle size distribution of WPINs. Effect of Na^+^ concentration on zeta potential (C) and particle size (D) of non-heat-induced and double-induced WPINs. ^a-d^ Different number superscripts represent significant differences in non-heat-induced and double-induced WPINs among different Na^+^ concentrates (*P* < 0.05).

Particle interaction is a critical factor to affect the emulsification performance (Ning et al., 2020). Investigating the zeta potential, particle size, and PDI of WPINs can provide valuable insights into the effects of double-induced on the formation of nanoparticles. The zeta potential, particle size, and PDI of WPINs are illustrated in Fig. 1. The effect of Na^+^ concentrations and heat-induced on the particle size distribution of WPINs is presented in Fig. 1 A and B. It can be observed that the particle size distribution for non-heat-induced WPINs is more uniform than that of double-induced WPINs at the same Na^+^ concentrations. The particle distribution of non-heat-induced WPINs shows a single peak, indicating a uniform particle distribution. However, the distribution of double-induced WPINs displays multiple peaks, suggesting an uneven particle distribution. The primary reason for this discrepancy appears to stem from a combination of the “salting-out” effect induced by Na^+^ along with heat treatment. Na^+^ may neutralize the negatively charged WPI particles, thereby reducing electrostatic repulsion and promoting increased aggregation through intermolecular disulfide bonds, thus forming a large network structure and causing the aggregation of WPI particles and formation of WPINs (Ning et al., 2020). Notably, There were no significant changes (*P* *<* *0.05*) observed in the PDI values for non-heat-induced WPINs at varying Na^+^ concentrations. However, the PDI of double-induced WPINs is consistent with the changes in particle size and increased with the increase of Na^+^ concentrations from 0 mM to 300 mM and subsequently decreased with the increase of Na^+^ concentrations from 300 mM to 500 mM. These findings further indicate that heat-induced in the existence of Na^+^ contributes to alteration in the particle size of WPINs. The zeta potential for both non-heat-induced and double-induced WPINs decreased as Na^+^ concentrations increased from 0 mM to 400 mM, remaining unchanged (*P* *>* 0.05) when Na^+^ concentrations increased from 400 mM to 500 mM (Fig. 1C). This phenomenon can also be contributed to a “salting-out” effect, where positively charged Na^+^ ions neutralize the negatively charged WPI particles, leading to a reduction in zeta potential (Ning et al., 2020). The particle size of WPINs is shown in Fig. 1D. It can be observed that the particle size of double-induced WPINs is significantly larger (*P* *<* 0.05) than that of non-heat-induced WPINs at the same Na^+^ concentrations. Furthermore, Na^+^ concentrations do no exert a significant influence (*P* *>* 0.05) on non-heat-induced WPINs. The particle size of non-heat-induced WPINs remains approximately 210 nm at various Na^+^ concentrations (0 mM to 500 mM). In contrast, the particle size of double-induced WPINs increases along with rising Na^+^ concentration from 0 mM to 300 mM, reaching a maximum 290 nm, before subsequently decreasing as Na^+^ concentration rises from 400 mM to 500 mM. These findings are consistent with those reported by Zhang et al. (2012). Who reported that the particle size of peanut protein significantly increased with salt ion concentration from 100 mM to 300 mM, after which it began to decrease as the salt ion concentration continued to rise. The findings indicated that the existence of salt ions and heat treatment could induce the polymerization of WPI particles, leading to the formation of nanoparticles. Liu & Tang, 2016a, Liu & Tang, 2016b,Liu & Tang, 2016c) also observed heat-induced aggregation of soy protein isolate (SPI) with increase of salt ion concentration from 0 mM to 300 mM at pH 7.0. Therefore, a Na^+^ concentration of 300 mM was chosen as the condition for heat-induced formation of WPINs.

### 3.2 Rheological properties of PEs

**Fig. 2:**
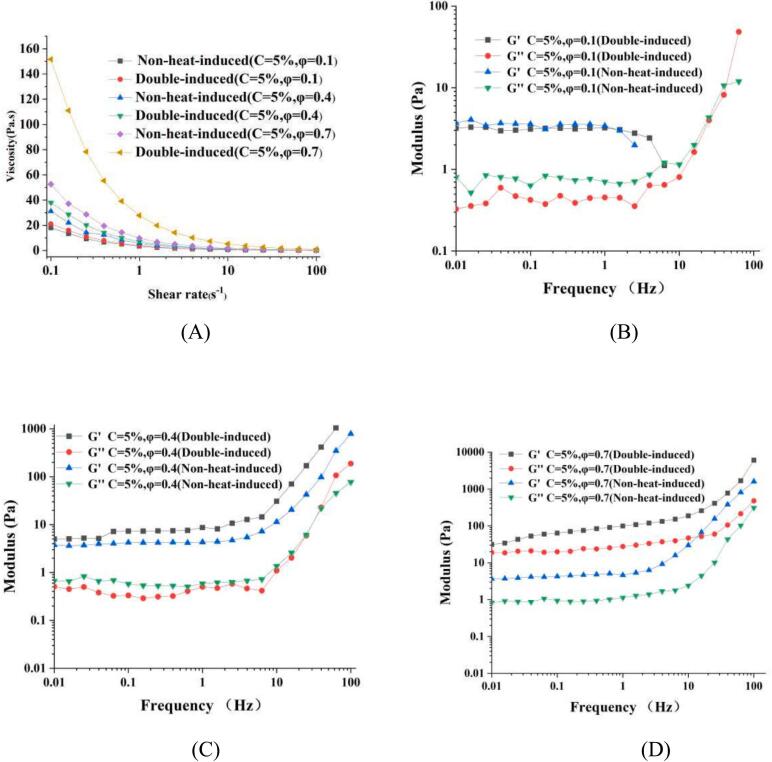
Apparent viscosity (A) and frequency sweep curves of PEs stabilized by non-heat-induced and double-induced WPINs (C = 5 %) at φ = 0.1 (B), 0.4 (C), and 0.7 (D).

The rheological properties of PEs with varying oil fractions stabilized by non-heat-induced and double-induced WPINs (C = 5 %) were investigated. As the increase in shear rate, the apparent viscosity of the PEs gradually decreased, demonstrating a pronounced shear-thinning behavior (Fig. 2A). This effect can be attributed to increasing disruption and alignment of structures in the emulsions as the shear rate increased, which resulted in a decrease in their resistance to flow (Zhang et al., 2012). At a concentration of 5 % WPINs, the apparent viscosity of PEs increased with higher oil fractions. The viscosity tended to increase when the oil fractions raised, which can be attributed to the flocculation of the oil droplets. Furthermore, PEs stabilized by double-induced WPINs exhibited higher apparent viscosity than that of non-heat-induced WPINs at the same φ value, especially for PEs with φ = 0.7 that were stabilized by double-induced WPINs. This effect can be explained that the ability of the double-induced WPINs to form a biopolymer network within the aqueous phase, as well as due to their ability to promote droplet aggregation at higher concentrations. The effects of oil fractions on the rheological properties of PEs were investigated through frequency sweeps (Fig. 2B). It was found that oil fraction significantly affects the modulus of PEs stabilized by WPINs. Notably, there is no significant difference in storage modulus (G′) between PEs stabilized by non-heat-induced and double-induced WPINs when the oil fraction is 0.1. The results indicated that PEs with φ = 0.1 are prone to flow and deformation when subjected to external forces; however, their physical properties, such as viscosity and stability, remain unchanged. With an increase of shear frequency, both the storage (G′) modulus and loss modulus (G″) of PEs increased at φ = 0.4 and 0.7. The G′ was significantly greater than G″, indicating that these PEs exhibited elastic behavior (Li et al., 2020). Furthermore, the G′ and G″ of PEs increased with rising oil fractions from 0.4 to 0.7, the G′ and G″ of PEs stabilized by double-induced WPINs at φ = 0.4 and 0.7 were greater than that of PEs stabilized by non-heat-induced WPINs. The findings suggest that double-induced WPINs could significantly promote the formation of PEs with superior elastic or solid-like properties at elevated φ levels compared to non-heat-induced WPINs (Li et al., 2020).

### 3.3 Effect of WPIN concentrations and oil fractions on the particle size of PEs

**Fig. 3:**
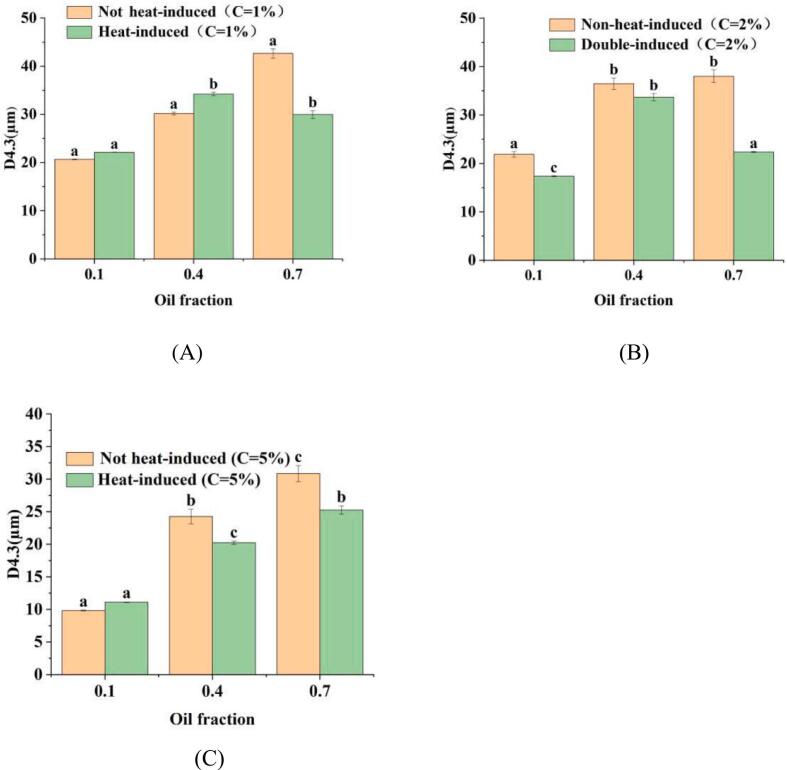
Particle size of PEs stabilized by WPINs at concentrations of 1 % (A), 2 % (B), and 5 % (C) with φ values ranging from 0.1 to 0.7. ^a-c^ Different number superscripts represent significant differences in PEs stabilized by double-induced and non-heat-induced WPINs (*P* < 0.05).

Both nanoparticle concentrations and oil fractions are closely related to the properties of PEs (Ning et al., 2020). The particle size of PEs stabilized by WPINs at varying concentrations and oil fractions φ is illustrated in Fig. 3. It was observed that the particle size of PEs stabilized by both non-heat-induced and double-induced WPINs increased as oil fractions increased from φ = 0.1 to 0.4 (Fig. 3). This finding aligns with the report by Ning et al. (2020), which indicated that droplet size increases with rising oil fractions at specific nanoparticle concentrations. This phenomenon can be attribute to the fact that an increase in oil fractions result in a great number of emulsion droplets, each processing smaller interfacial areas, thereby necessitating more available WPINs for coverage over these droplets, more emulsion droplets were expected to more WPINs adsorption. Thus, it follows that the particle size of PEs increased as the oil fractions increase. However, it is noteworthy that the particle size of PEs stabilized by double-induced WPINs exhibited a significant decrease (*P* *<* 0.05) at φ = 0.7 when compared to φ = 0.1 and 0.4 at C = 1 % and 2 % (Fig. 3A, B). Furthermore, at C = 5 %, the particle size of PEs stabilized by both non-heat-induced and double-induced WPINs were significantly lower (*P* *<* 0.05) than those stabilized by their counterparts at C = 1 % and C = 2 % (Fig. 3 C). The results illustrate that at high concentrations of WPINs, a relatively high amount of WPINs were absorbed to the surface of oil droplets, thereby enhancing their stability, as the WPINs concentration increases, smaller droplet sizes are formed. When comparing PEs stabilized by double-induced WPINs at the same oil fractions and WPINs concentrations (excluding 1 % WPINs concentration), it was found that the particle size in the former case is significantly lower (*P* *<* 0.05). This indicates that PEs stabilized by double-induced WPINs can significantly reduce the particle size. These findings can be attributed primarily to the fact that double-induced WPINs form a dense and rigid network structure, resulting a relatively thick protective film on the surface of oil droplets within PEs. This structure enhancement contributes to improve stability of PEs and prevent aggregation of oil droplets (Ning et al., 2020).

### 3.4 Microstructure of PEs prepared by WPINs and oil fractions

**Fig. 4:**
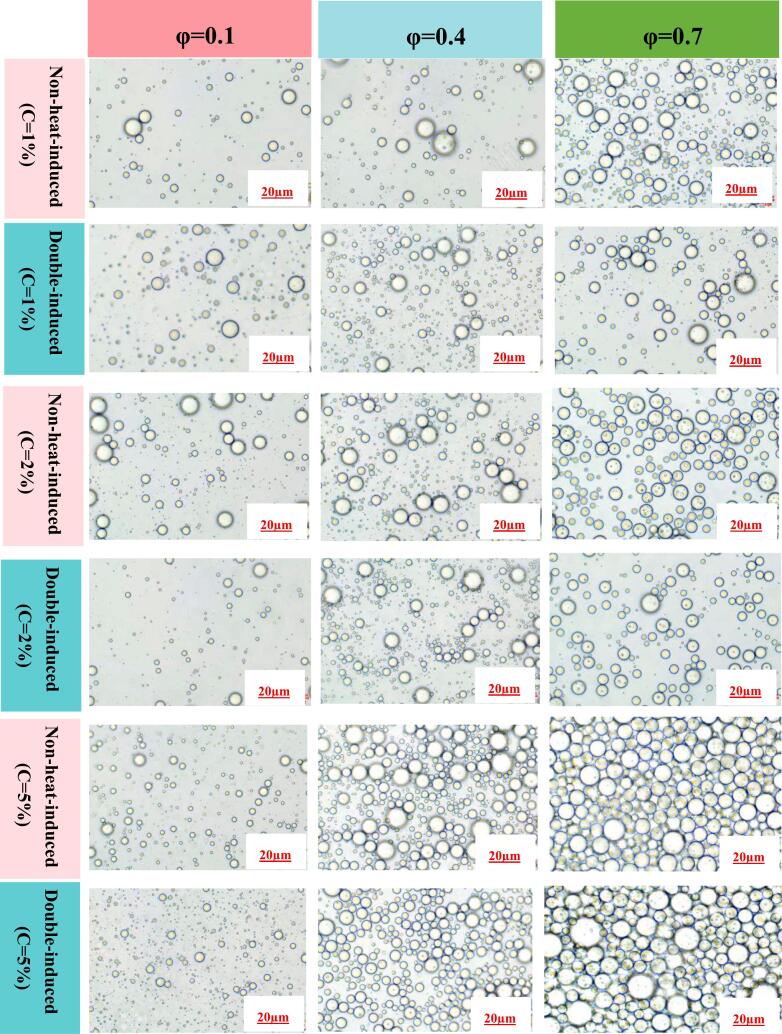
Microstructure of PEs prepared by varying WPIN concentrations (1–5 %) and different φ values (φ = 0.1–0.7).

**Fig. 5:**
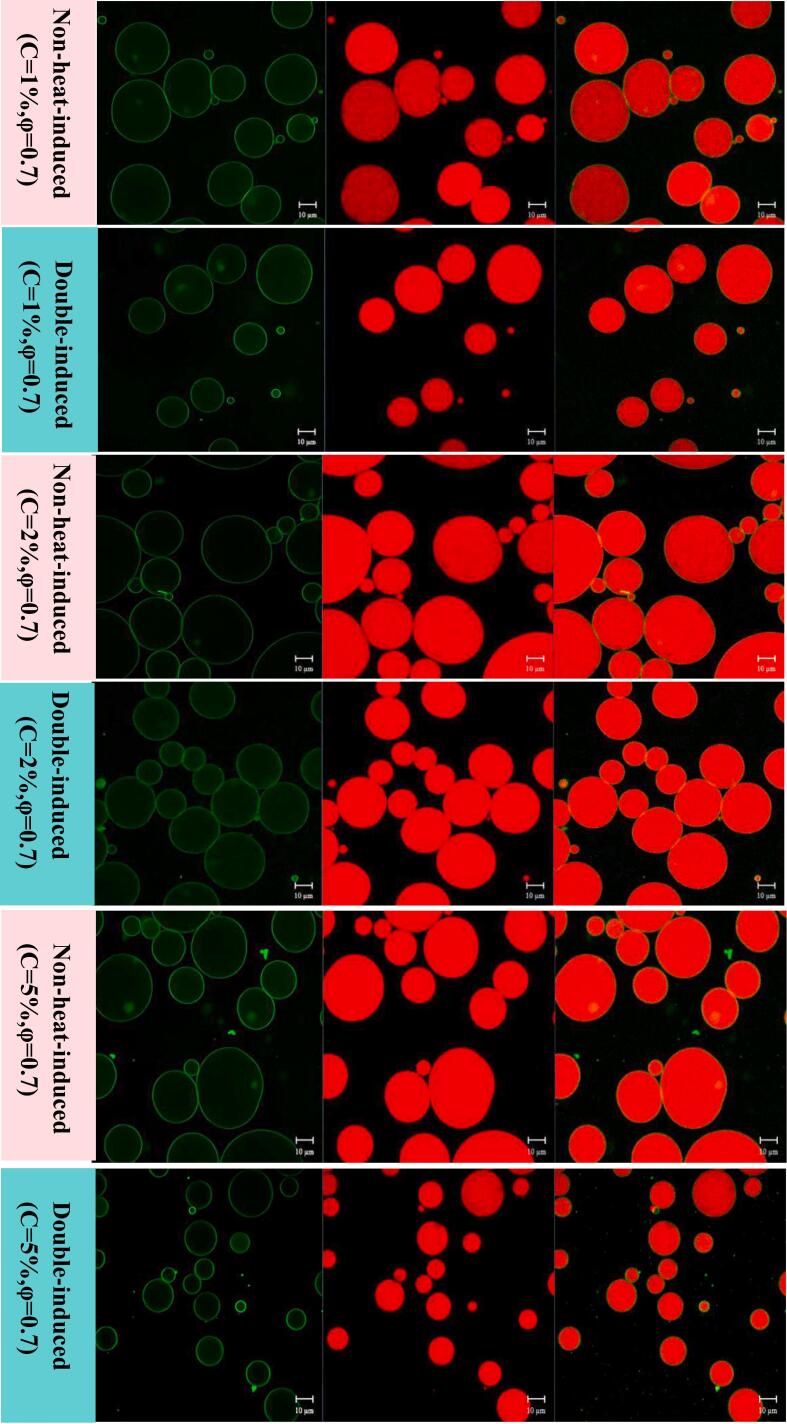
CLSM of WPINs stabilized PEs at specific φ = 0.7 and varying Na^+^ concentrates of 1–5 %.

An optical microscopy was used to observe the PEs morphologies (Fig. 4). The microstructural images provided visual confirmation of PEs. Notably, PEs with high oil fractions exhibited a significant number of oil droplets and a relatively inhomogeneous distribution (Fig. 4). Previous studies have indicated that the size of PEs stabilized by zein/carboxymethyl dextrin nanoparticles (Meng et al., 2020) and novel pea protein isolate nanoparticles (Li et al., 2022) increases with as the volume fraction of oil phase rises. This phenomenon could be explained by the fact that the reduction in available protein particles capable of adsorbing at the oil-water interface, which consequently leads to an increase in PE size with higher oil volume fractions (Wang, Chen, et al., 2024; Wang, Lin, et al., 2024). The oil droplets of PEs stabilized by double-induced WPINs exhibited a more homogeneous distribution compare to those stabilized by non-heat-induced WPINs at identical oil fractions and WPIN concentrations. Additionally, the oil droplets of PEs stabilized by both non-heat-induced and double-induced WPINs exhibited an increasingly homogeneous distribution as the WPIN concentrations rose while maintaining the same oil fraction. CLSM images of the PEs (φ = 0.7) at varying WPIN concentrations are shown in Fig. 5. The oil droplets of PEs stabilized by double-induced WPINs exhibited a more uniform distribution than those stabilized by non-heat-induced WPINs. Furthermore, the oil droplets of PEs stabilized by doublet-induced WPINs showed a decreasing trend in size with increasing WPIN concentrations.

### 3.5 Stability of PEs

#### 3.5.1 Storage stability

**Fig. 6:**
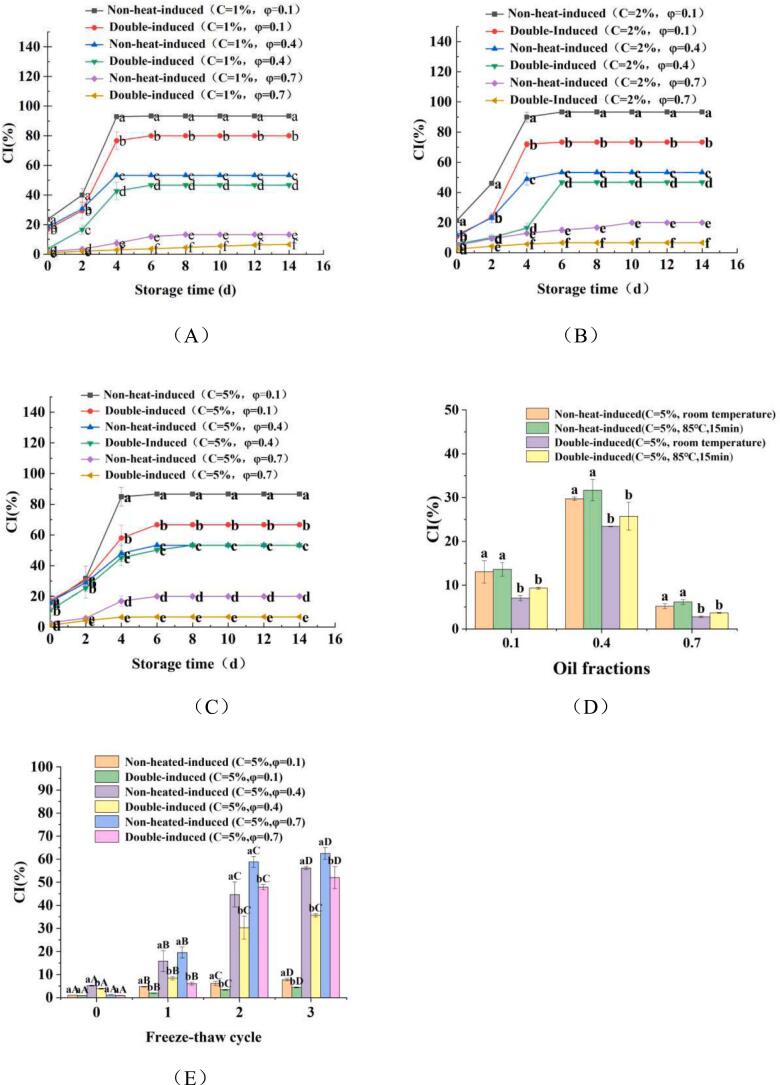
CI% of the PEs stabilized by WPINs at φ values ranging from 0.1 to 0.7, with varying Na^+^ concentrates of 1.0 % (A), 2.0 % (B) and 5.0 % (C) during 14 d storage time. CI% of the PEs stabilized by 5 % WPINs at φ values ranging from 0.1 to 0.7 during thermal treatment (D) and freeze-thaw cycle (E). ^a-b^ Different number superscripts represent significant differences between PEs stabilized by non-heat-induced and double-induced WPINs at the same freeze-thaw cycle (*P* < 0.05). ^A-B^ Different number superscripts represent significant differences between PEs stabilized by non-heat-induced and double-induced WPINs at different freeze-thaw cycles (*P* < 0.05).

If the PE is unstable during storage, its fat will float up because of its light density, resulting in the separation of oil and serum. The cream separation index (CI) is defined as the percentage of the height of the clear emulsion relative to the total height of the whole emulsion. A smaller CI indicates a more stable emulsion; thus, CI serve as an effective metric for evaluating PE stability during storage. Fig. 6. shows the stability of PEs stabilized by varying concentrations of WPINs, derived from non-heat-induced and double-induced process, along with different oil fractions after 14 days of storage. It can be found that the impact of oil fractions on PE stability was significantly higher (*P* *<* 0.05) than that of WPINs concentration. Regardless of protein concentration, Freshly prepared PEs at oil fractions of 0.1 and 0.4 exhibited rapid creaming behavior across all emulsions, indicating creaming instability. During the initial 4 days, there was a swift increase in CI%, which subsequently slowed down over time until it reached a near-constant value. Notably, PEs prepared with φ = 0.7 presented a more stable trend compared to those prepared with φ = 0.1 and 0.4 at the same WPIN concentration (Fig. 6 A, B, C). The results are consistent with those reported by Ning et al. (2020), who found that the stability of emulsions increased with a higher oil fraction. When the oil fractions increase, higher density of particles in aqueous phase would anchor more particles onto the interface, thus contributed to stabilize higher interfacial area during the limited coalescence process.Otherwise, The CI% of PEs stabilized by double-induced WPINs is significantly lower (*P* *<* 0.05) compared to PEs stabilized by non-heat-induced WPINs at the same φ. This difference can be attribute to the tendency of double-induced WPINs to form a gel-like network, which acts as an interfacial film covering the interface of PEs and thereby limiting the coalescence process. According to Ning et al. (2020), this enhancement in creaming stability is largely contributed to the formation of a gel-like network. When the oil fraction increases, the emulsion became gel-like and more stable. The formation of the gel-like network by WPINs plays an important role in stabilizing the PEs. Liu and Tang (2013) also evaluated the PEs stabilized by soy protein nanoparticles and found that the PEs presented more stable (CI = 70 %) after 35 days of storage when the C = 2.0 %, φ = 0.6.

#### 3.5.2 Thermal stability

The thermal stability of PEs with different oil fractions (φ = 0.1, 0.4, and 0.7), stabilized by non-heat-induced and double-induced WPINs (C = 5 %) was investigated. A control temperature of 25 °Cwas employed, and the investigation index used was the CI% (Fig. 6D). The results indicated that PEs stabilized by both non-heat-induced and double-induced WPINs (C = 5 %) exhibited the lowest thermal stability at an oil fraction of φ = 0.4. The lower CI% observed in PEs with φ = 0.1 can be attributed to its reduced oil content, while PEs with φ = 0.7 presented high thermal stability (Fig. 6D). It may be due to the fact that a higher density of droplets being tightly packed,which restricts the droplet movement and enhances resistance to deformation (Wei & Huang, 2019). Otherwise, rheological property analyses revealed that PEs with elevated φ displayed increased apparent viscosity and gel-like structure; these characteristics contribute positively to overall stability of PEs. Notably, There were no significant difference (*P* *>* *0.05*) in CI% between thermally treated and non-thermally treated PEs sharing the same φ value. This finding suggests that both stabilized methods resulted in high thermal stability for the PEs. It was found that the CI% for PEs stabilized by double-induced WPINs (C = 5 %) is significantly lower (*P <* 0.05) than that of non-heated induced WPINs (Fig. 6D). These results indicated that PEs stabilized by double-induced WPINs exhibit superior thermal stability than that of non-heat-induced WPINs. It is also possible that PEs stabilized by double-induced WPINs exhibited higher apparent viscosity and a more compact gel-like structure. These findings are consistent with the microstructure of PEs stabilized by both non-heat-induced and double-induced WPINs under heat treatment conditions (Fig. S2). There was no significant difference in microstructure of PEs stabilized by non-heat-induced and double-induced WPINs at the same φ. It further indicated that PEs stabilized by both non-heat-induced and double-induced WPINs posses high thermal stability. When the oil fraction is 0.4. PEs stabilized by non-heat-induced and double-induced WPINs underwent heat treatment, resulting in a large droplet distribution.

#### 3.5.3 Freeze-thaw stability

**Fig. 7:**
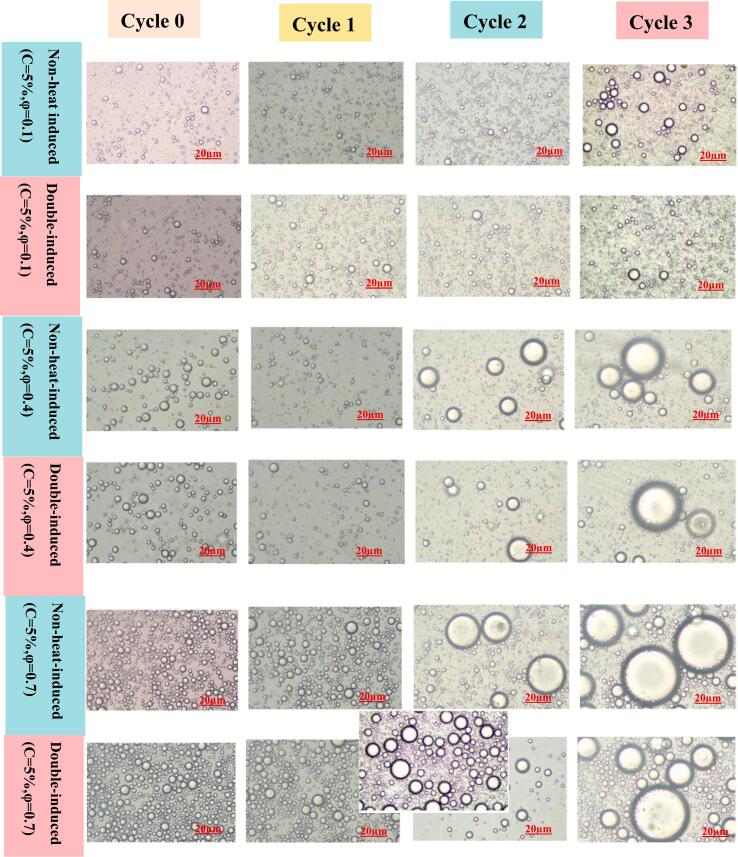
Microstructure of PEs (φ = 0.1–0.7) prepared with 5 % WPINs concentration during the freeze-thaw cycle.

As shown in Fig. 6E. freeze-thaw treatment significantly affects the stability of PEs stabilized by non-heat-induced and double-induced WPINs. The CI% of PEs stabilized by both non-heat-induced and double-induced WPINs increased with the rise in φ after three freeze-thaw cycles (Fig. 6E). The structure of PEs observed by optical microscopy corroborated these findings. During the freeze-thaw process, the droplets of PEs significantly increase in size (Fig. 7). It indicated that PEs stabilized by non-heat-induced and double-induced WPINs exhibited instability under freeze-thaw condition. This phenomenon can be attribute to the formation of ice crystals within the continuous phase of the PEs during freezing, which squeezed the droplets and promoted droplet movement and mutual aggregation. An increase in droplet quantity exacerbates this squeezing effect (Zhu et al., 2017). Otherwise, the ice crystals pierced the interfacial membrane of some droplets, which led to the merging of adjacent droplets and the formation of larger droplets. Furthermore,The fat phase might solidify at low temperature, and the fabricated fat crystals could penetrate and disrupt the interfacial layer around the oil droplets, resulting in full coalescence after thawing (Lin et al., 2021; Zhu et al., 2017). The CI% of PEs stabilized by double-induced WPINs was significantly lower (*P* *<* 0.05) than that for those stabilized by non-heat-induced WPINs at the same φ during freeze-thaw cycle (Fig. 6E). This observation could be explained by the higher apparent viscosity exhibited by PEs stabilized with double-induced WPINs; high-viscosity emulsions are more effective at resisting ice crystals formation and emulsion particles aggregation during freezing and thawing process.

### 3.6 Digestion properties of PEs stabilized by non-heat-induced and double-induced WPINs

**Fig. 8:**
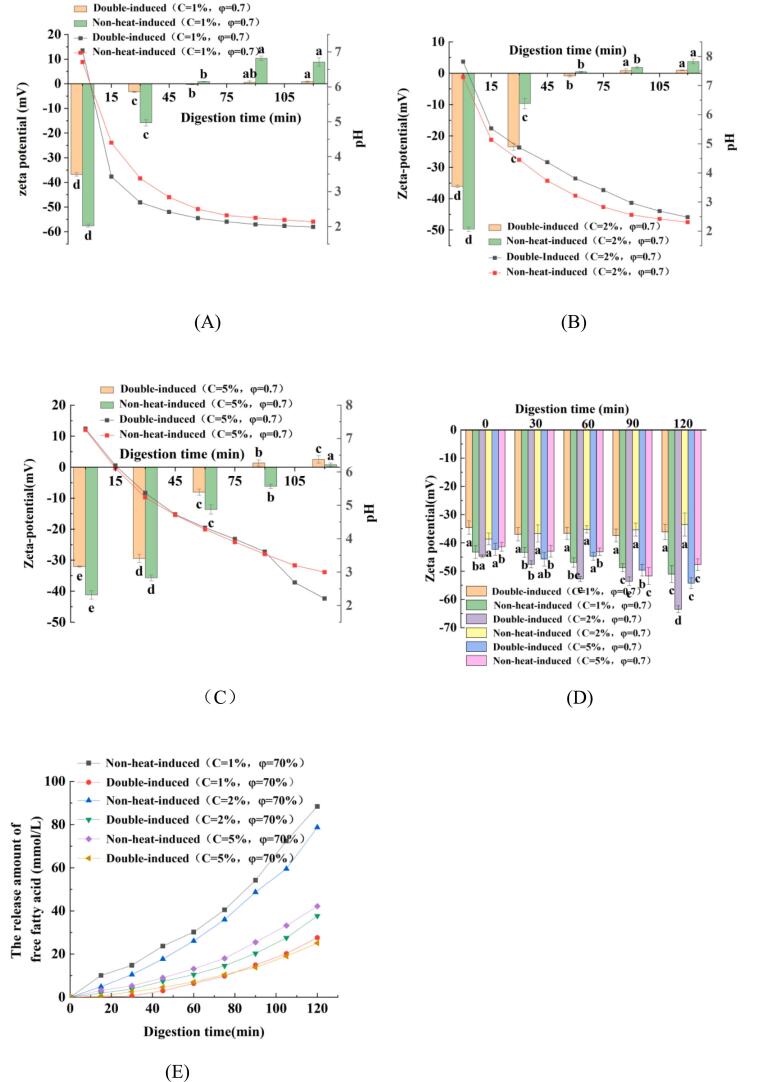
Zeta potential for the PEs stabilized by WPINs at specific φ = 0.7, with varying Na^+^ concentrates of 1.0 % (A), 2.0 % (B), and 5.0 % (C) during the gastric and intestinal (D) digestive process. The release amount of free fatty acid (E) for the PEs during the intestinal digestive process. ^a-c^ Different number superscripts represent significant differences in PEs among different digestive time (*P* < 0.05).

Lipid digestion involves in bio-interfacial process, which is determined by the absorption of lipase-bile salts complex on the surface of oil droplets (Sarkar et al., 2019). The changes in zeta-potential of PEs with varying oil fractions stabilized by non-heat-induced and double-induced WPINs are illustrated in Fig. 8A, B, and C. During gastric digestion, the zeta-potential of PEs exhibits a consistent trend; it transitions from positive to negative charge as digestion time increase. Notably, these changes in zeta potential depended on the changes in pH. An interesting phenomenon is that the lower positive charge occurred in PEs stabilized by non-heat-induced and double-induced WPINs after 60 min gastric digestion time. Theses phenomenon could be explained as follows: First, PEs possess a significant number of negative charges before entering the stomach for digestion, changing from negative charges to positive charges requires a large number of H^+^ to realize it. Second, during gastric digestion, pH decreases gradually pH gradually across due to an increase of H^+^ (Fig. 8A, B, and C). The pH of emulsions decreased to below 2.5 after approximately 60 min of gastric digestion. The third reason pertains to the structure of PEs stabilized by WPINs. During gastric digestion, emulsion gels are formed from PEs, resulting in a more compact gel-like network with smaller pores and trapping the oil droplets in them, thus hindering the adsorption of bile salts and causing the low positive charge (Li et al., 2021). During intestinal digestion, digestive juices carry a significant number of negative charges (Fig. 8D). This phenomenon could be explained as follows. i) protein aggregations were hydrolyzed by protease in SIF and produced peptides; ii) lipids were hydrolyzed by lipase in SIF and produced free fatty acids (FFAs); iii) these small molecular substances, such as peptides, FFAs and bile salts from SIF were absorbed on the interface of the fat droplets and caused the increase of the negative charges (McClements & Li, 2010). The release of FFAs represents the speed of lipid digestion in the SIF phase. Considering that PEs present stable at φ = 0.7, PEs with special φ = 0.7 were used to investigate the intestinal digestion properties. Fig. 8E showed FFAs release properties for PEs with φ = 0.7 stabilized by different WPIN concentrations during intestinal digestion. The FFA release amount became slower with the increase in WPIN concentrations. Otherwise, it was also found that the FFAs release of PEs stabilized by double-induced WPINs was slower than that of non-heat-induced WPINs (Fig. 8E). The low release amount of FFAs indicates that lipid digestion was inhibited. In short, several factors that influence the digestion and the final release of PEs include droplet size, interface compositions and structure, and the viscosity of the aqueous phase of emulsions (Lu et al., 2017; Mao et al., 2014; Qian et al., 2012; Salvia-Trujillo et al., 2017). The following reasons could be used to explain the low release amount of FFAs from PE stabilized by double-induced WPINs. First and foremost, a thicker interfacial film was formed when PEs are stabilized by double-induced WPINs, the thickness of this interfacial film increases with higher concentration of Na^+^. The thicker interfacial prevents the bile salts and lipases from effectively contacting the lipid droplets, resulting in a lower release of FFAs (Li et al., 2021; Liu & Tang, 2016a; Liu & Tang, 2016b; Liu & Tang, 2016c). Second, the electrostatic repulse between bile salts and droplets hinders the adsorption of bile salts onto the droplet surface (Wei & Huang, 2019). Third, the digestive enzymes are also key factors affecting lipid digestion; specifically, the microstructure of PEs stabilized by double-induced WPINs impedes the diffusion of digestive enzymes, thereby preventing the lipase from hydrolyzing the lipid droplets (Chen et al., 2022). Fourth, PEs stabilized by double-induced WPINs increased the viscosity of the aqueous phase and hindered the pancreatic enzyme access the fat droplets, leading to a slower release of FFA from emulsion droplets. Furthermore, the adsorption of bile salt on the droplet surface plays an important part in lipid digesting (Hu et al., 2021). The limited adsorption of bile salts reduces opportunities for lipase to interact with lipids, thereby the lipid digestion was delayed and caused the low release of FFAs. These findings provided valuable insights into controlling and prolonging the intestinal release of bioactive substances and nutraceuticals.

## 4 Conclusion

In summary, the particle size of double-induced WPINs is significantly larger than that of non-heat-induced WPINs at the same Na^+^ concentrations. Under identical oil fraction and WPIN concentration, the particle size of PE stabilized by double-induced WPINs is notably smaller compared to that stabilized by non-heat-induced WPINs. Among various oil fractions, PE prepared with oil fraction φ = 0.7 presented superior stability relative to other formulations. Furthermore, PE stabilized by double-induced WPINs presents higher storage stability, thermal stability, and freeze-thaw stability than that of non-heat-induced WPINs at the same φ. The release rate of FFAs from PE stabilized by double-induced WPINs was slower than that of non-heat-induced WPINs. To the authors' knowledge, these findings would be beneficial for the exploration of food-grade emulsion formulations with excellent stability and digestive properties. Consequently, this research has significant implications for expanding the application of protein-based nanoparticles as Pickering-type stabilizers in the food industry.

## CRediT authorship contribution statement

**Shenghua He:** Writing – original draft, Investigation, Funding acquisition, Formal analysis, Data curation, Conceptualization. **Yonghui Wang:** Resources, Methodology, Formal analysis, Data curation, Conceptualization. **Guanghui Li:** Writing – review & editing, Software, Methodology, Formal analysis, Data curation. **Xueli Gao:** Software, Methodology, Formal analysis, Data curation. **Zhiyan Chen:** Methodology, Investigation, Formal analysis, Data curation, Conceptualization. **Weiyun Guo:** Writing – review & editing, Project administration, Funding acquisition, Formal analysis, Data curation, Conceptualization. **Jihong Huang:** Software, Resources, Funding acquisition, Conceptualization.

## Declaration of competing interest

The authors declare that they have no known competing financial interests or personal relationships that could have appeared to influence the work reported in this paper.

## Data Availability

The data that has been used is confidential.

## Appendix A Supplementary data

Fig. S1. Microstructure of PEs with φ values (φ = 0.1-0.7) prepared by 5% WPINs concentration during thermal treatment. Image 1
