# Supplementary material for: Effect of double-induced on whey protein isolate nanoparticle formation and stabilized food-grade Pickering emulsions: Stability and gastrointestinal digestion
Source: Food Chem X. 2025 Feb 6;26:102221. doi: 10.1016/j.fochx.2025.102221 (PMC11849594; doi:10.1016/j.fochx.2025.102221)
Supplement: Supplementary file 1 — Fig. S1. Microstructure of PEs with φ values (φ = 0.1-0.7) prepared by 5% WPINs concentration during thermal treatment. [file mmc1.docx]

**Supplementary data**

**Double induced**

**(Heat treatment)**

**Double induced**

**(Non-heat treatment)**

**Non-heat induced**

**(Heat treatment)**

**Non-heat induced**

**(Non-heat treatment)**


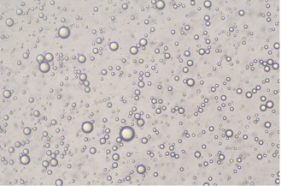

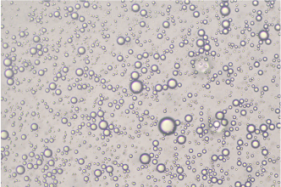

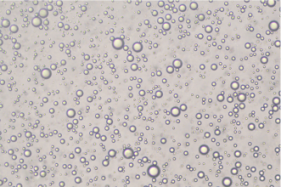

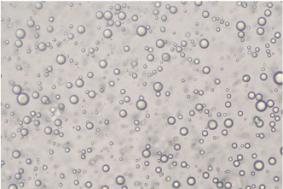


**20µm**

**20µm**

**20µm**

**20µm**

**C=5%,φ=0.1)**


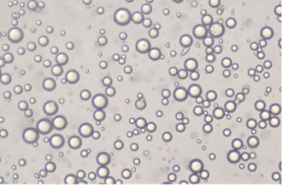

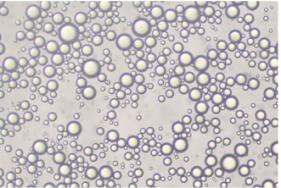

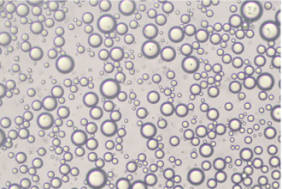

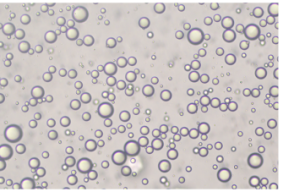


**20µm**

**20µm**

**20µm**

**20µm**

**(C=5%,φ=0.4)**


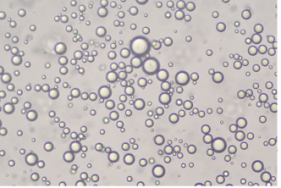

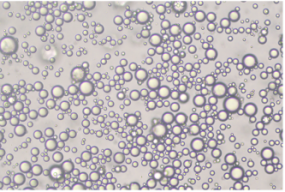

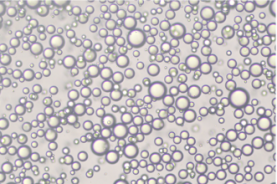

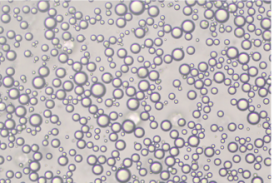


**20µm**

**20µm**

**20µm**

**20µm**

**(C=5%,φ=0.7)**

Fig. S2. Microstructure of PEs with φ values (φ = 0.1-0.7) prepared by 5% WPINs concentration during thermal treatment.

There is no significant difference in microstructure for PEs stabilized by non-heat-induced and double-induced WPINs with the same φ between non-heat treatment and heat treatment. It further indicated that PEs stabilized by non-heat-induced and double-induced WPINs had high thermal stability. When the oil fraction is 0.4. PEs stabilized by non-heat-induced and double-induced WPINs underwent heat treat and had big droplets distribution.
